# Clinical utility of tibial motor and sensory nerve conduction studies with motor recording from the flexor hallucis brevis: a methodological and reliability study

**DOI:** 10.1186/1757-1146-4-14

**Published:** 2011-05-24

**Authors:** Kathleen M Galloway, Mark E Lester, Rachel K Evans

**Affiliations:** 1 United States Army Research Institute of Environmental Medicine, Natick, MA, USA; 2 Oakland University, Rochester, MI, USA

## Abstract

**Background:**

Standard tibial motor nerve conduction measures are established with recording from the abductor hallucis. This technique is often technically challenging and clinicians have difficulty interpreting the information particularly in the short segment needed to assess focal tibial nerve entrapment at the medial ankle as occurs in posterior tarsal tunnel syndrome. The flexor hallucis brevis (FHB) has been described as an alternative site for recording tibial nerve function in those with posterior tarsal tunnel syndrome. Normative data has not been established for this technique. This pilot study describes the technique in detail. In addition we provide reference values for medial and lateral plantar orthodromic sensory measures and assessed intrarater reliability for all measures.

**Methods:**

Eighty healthy female participants took part, and 39 returned for serial testing at 4 time points. Mean values ± SD were recorded for nerve conduction measures, and coefficient of variation as well as intraclass correlation coefficients (ICC) were calculated.

**Results:**

Motor latency, amplitude and velocity values for the FHB were 4.1 ± 0.9 msec, 8.0 ± 3.0 mV and 45.6 ± 3.4 m/s, respectively. Sensory latencies, amplitudes, and velocities, respectively, were 2.8 ± 0.3 msec, 26.7 ± 10.1 μV, and 41.4 ± 3.5 m/s for the medial plantar nerve and 3.2 ± 0.5 msec, 13.3 ± 4.7 μV, and 44.3 ± 4.0 msec for the lateral plantar nerve. All values demonstrated significant ICC values (*P *≤ 0.007).

**Conclusion:**

Motor recording from the FHB provides technically clear waveforms that allow for an improved ability to assess tibial nerve function in the short segments used to assess tarsal tunnel syndrome. The reported means will begin to establish normal values for this technique.

## Background

Posterior tarsal tunnel syndrome is a clinical description of tibial nerve compression at the ankle, as the tibial nerve passes through the tarsal tunnel posterior to the medial malleolus. The tibial nerve then branches into the medial plantar and lateral plantar nerves either at the level of the tarsal tunnel or immediately distal as the branches enter the foot. In this same region, there are calcaneal branches from the tibial nerve that supply sensation to the inferior aspect of the heel [1]. Pes planus, activity level and lower extremity edema are factors that have been proposed to create posterior tarsal tunnel syndrome [1-5]. There are also reports of posterior tarsal tunnel syndrome related to foot deformities, [6,7] tumor and varicosities [2,8-11].

Posterior tarsal tunnel syndrome may present with selective involvement of the medial plantar, lateral plantar, and/or calcaneal branches of the tibial nerve [2,6,12]. Clinical presentation of posterior tarsal tunnel syndrome often includes burning in the sole of the foot and may include pain in the inferior calcaneus. Nerve conduction and electromyographic studies are collectively referred to as electrodiagnostic studies which are considered to be definitive objective tests for posterior tarsal tunnel syndrome [2]. The complete electrodiagnostic exam includes evaluation for other associated pathologies that may produce burning in the sole of the foot to include lumbosacral radiculopathy and polyneuropathy. Once these pathologies have been evaluated, the examiner assesses the conduction of the tibial motor and sensory nerve branches across the tarsal tunnel [13].

The most common recording techniques for the medial plantar motor branch of the tibial nerve (S2-S3) involve recording over the motor point of the abductor hallucis muscle [13]. Proper electrode placement over the motor point of the muscle ensures a waveform with a clear initial negative deflection from baseline. The latency or time measure of the motor nerve is recorded at the point the waveform is initiated. Precise distance and latency measurements are needed to ensure accurate nerve conduction values [2].

Another muscle innervated by the medial plantar motor branch of the tibial nerve (S2-S3) is the flexor hallucis brevis (FHB) muscle. Recording from the FHB may then give similar information to abductor hallucis. The FHB has its proximal attachment over the plantar aspect of the cuboid and lateral cuneiform bones, with distal attachment on both the medial and lateral sides of the proximal phalanx of the hallux. The FHB acts to flex the proximal phalanx of digit one. The musculature of the plantar foot is often described in layers. There are four layers with layer one being the most superficial and layer four being the deepest layer. The abductor hallucis, flexor digitorum brevis and abductor digiti minimi muscles are described in layer one. The tendons of the flexor hallucis longus and flexor digitorum longus with associated lumbricals and quadratus plantae are located in layer two. The flexor hallucis brevis is described in layer three with the more laterally placed adductor hallucis and flexor digiti minimi brevis muscles. The dorsal and plantar interosseous muscles are deepest and located in layer four [1].

Normative values have been published for the tibial motor nerve with recording from the abductor hallucis, however recording from the abductor hallucis sometimes leads to technical challenges [14-17]. In fact, Del Toro et al indicate that there may be multiple motor points in the abductor hallucis muscle, leading to difficulty establishing a clear initial negative deflection from baseline [17]. It becomes challenging to accurately determine nerve conduction velocity without a clear onset of the motor response. This is particularly difficult when assessing a short segment such as tibial motor nerve conduction velocity across the tarsal tunnel [18]. A minor deviation in latency marker placement can have a large impact on the conduction velocity calculated in the tarsal tunnel segment. This technical difficulty has led some clinicians to conclude that nerve conduction studies are not highly sensitive in detecting posterior tarsal tunnel syndrome, and some make the diagnosis of posterior tarsal tunnel syndrome without objective nerve conduction or electromyographic findings [7-9]. Powell reports a false negative result of 9.5% with electrodiagnostic studies in posterior tarsal tunnel syndrome [19].

Tibial motor nerve recording from the flexor hallucis brevis muscle has been referenced as an alternative to abductor hallucis recording [20]. This is clinically useful since the FHB, like the abductor hallucis is supplied by the medial plantar nerve branch of the tibial nerve [1]. Felsenthal et al reported recording a medial plantar motor latency response from the flexor hallucis brevis in a trans-tarsal technique with 20 normal young adult participants [20]. It is possible that recording from the FHB may be valuable especially when recording from the abductor hallucis has become problematic. There were no additional reported normative values or reliability studies for recording from the FHB identified.

Tibial sensory conduction values also need to be evaluated in patients with possible tarsal tunnel syndrome complaints [13,20]. Updated normative values have been published for both antidromic [21,22] and orthodromic medial and lateral plantar sensory techniques [23,24]. There are, however, few well controlled studies using adequate sample sizes to establish normative data for tibial sensory nerve conduction values. Additionally, the reliability of these measures has not been well established. Belen et al anecdotally reported good reliability with an N of 41 participants using an orthodromic technique [23]. In addition, Loseth et al reported acceptable intraobserver agreement of a single investigator performing two separate medial plantar sensory conduction studies on 22 healthy participants [25]. These authors found no statistically significant difference between amplitude and velocity measurements. They further reported interobserver agreement between two observers using the same technique on 46 participants and found no significant differences between measurements [25].

The purpose of our study was to establish normative values for motor studies recorded from the FHB, and to provide additional reference values for medial and lateral plantar orthodromic sensory measures. We also sought to assess intrarater reliability for all techniques.

## Methods

To establish normal values, 80 women between the ages of 18 and 35 were recruited from the student and community populations surrounding a university in Southeastern Michigan. A sub-group of 40 participants were available to participate in serial collections at 2, 4, and 6 months following the initial session to establish intra-rater reliability of the measures. All participants included in this analysis were participants in a larger study of the effects of whole body vibration on bone density in young women. The results of this larger project are not yet published. The initial 80 participants had nerve conductions completed prior to participating in any whole body vibration intervention, while the 40 participants who returned for serial nerve conduction studies were control participants for this same study. Each participant read and signed a written informed consent document before proceeding with the study. A screening questionnaire was used to determine whether potential participants had a history of back pain, diabetes, peripheral neuropathy, polyneuropathy, planned pregnancy, kidney disease, foot pain, history of leg or foot injury, cardiac pathology, current pregnancy, malignancy, balance disorders or vascular pathology. Potential participants with a history of the aforementioned conditions were excluded, as were individuals who participated in weight bearing sporting activities more than three times per week. Participants not excluded by medical history or activity level were allowed to enroll in the study. The institutional review boards at Oakland University, Rochester, MI, and the U.S. Army Research Institute of Environmental Medicine, Natick, MA approved the protocol and study design.

Participants reported to the research laboratory in athletic attire (shorts and t-shirts), with their shoes removed. Height (cm) was measured using a stadiometer, and body weight (kg) was recorded using a digital scale. A body mass index was calculated.

Motor and sensory nerve conduction values (latency, amplitude and velocity) were assessed on the non-dominant leg using the Cadwell Sierra LT EMG machine (Cadwell Laboratories, Inc., Kennewick, WA). The participant was asked to lie in a supine position on a treatment table. Moist hot packs were applied to the foot in order to maintain skin temperature at or above 29 degrees Celsius [16,26,27]. We established 29 degrees Celsius as a lower limit for surface skin temperature as in the Felsenthal study [20], however all participants received a hot pack prior to examination and exceeded this value. The hot pack was removed immediately prior to nerve conduction evaluation. Skin temperature was monitored at the dorsum of the foot using a digital surface thermometer model 100A (VWR Scientific, West Chester, PA 19380) throughout the examination to maintain the temperature between 30 and 32 degrees Celsius.

**Figure 1:**
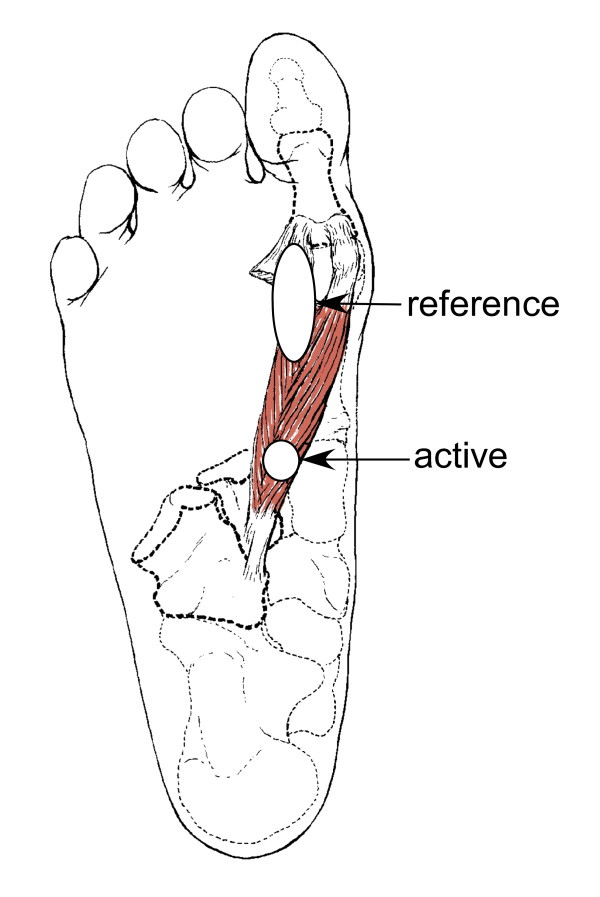
**Recording sites for tibial motor study**. Active and reference recording sites for the tibial motor study, located over the lateral head of the flexor hallucis brevis muscle on the plantar surface of the foot, with the reference electrode distal.

The tibial motor study was conducted with placement of the active recording electrode over the motor point of the flexor hallucis brevis muscle, and the reference electrode 3 cm distal to the active recording electrode. Placement of the active recording electrode was approximately 2 cm lateral and 3-4 cm distal to the navicular tuberosity over the lateral head of the flexor hallucis brevis, immediately to the lateral side of the flexor hallucis longus tendon. The reference electrode was placed 3 cm distal (Figure 1).

The distal stimulation site for the tibial motor nerve was located 12 cm proximal to the reference electrode posterior to the medial malleolus, however the recording cathode was repositioned if a clear waveform was not recorded. Separate supramaximal stimulations were delivered posterior to the medial malleolus and at the popliteal fossa using a square monophasic waveform, a pulse width varying between 0.1 and 1.0 msec, and intensity up to 100 mA. The skin was marked at each stimulation site and the distance between them measured using a standard tape measure. Motor latencies were recorded from the waveform onset. Motor amplitude was measured from the waveform baseline to the waveform peak.

The tibial (medial and lateral plantar) sensory study was completed by first placing the active recording electrode posterior to the medial malleolus, and the reference electrode 3 cm proximal to the active recording electrode. The stimulation point for the medial plantar sensory nerve was placed between the first and second metatarsals 12 cm distal to the recording electrode, however if an adequate waveform could not be elicited, the stimulating and/or recording electrodes were repositioned. The stimulation point for the lateral plantar sensory nerve was between the fourth and fifth metatarsals 14 cm distal to the active recording electrode, again if an adequate waveform could not be elicited; the stimulating and/or recording electrodes were repositioned. Latency was measured from the peak of the initial negative deflection and used to calculate conduction velocity. Amplitude was measured from waveform peak to peak.

Descriptive statistics were used to present mean and standard deviation values for all parameters at baseline and at subsequent 8 week, 16 week and 24 week data collection sessions. The root mean square error, standard error of the measurement, and minimal detectable change values were also calculated for each parameter to establish variability and determine a clinically meaningful detectable difference. Two forms of the coefficient of variation (CV) were calculated to include the variable coefficient of variation to establish between participant variability at a given time point. In addition a model coefficient of variation was calculated by dividing the mean of the absolute value of the root mean square by the group mean to establish within participant variability over time. We examined reliability for serial measures using an ANOVA technique to assure there were no significant differences between time points. Intraclass correlations (ICC) (model 3,1) were calculated to determine reliability between all time points for the nerve conduction measures. Correlation coefficient values greater than 0.5 were considered to indicate good reliability [28]. Significance was set at *P *≤ 0.05. Data were analyzed using SPSS (Statistical Package for the Social Sciences, version 16.0, SPSS Inc., Chicago, IL).

## Results

Motor amplitude and velocity parameters as well as the medial and lateral plantar sensory nerve conduction values and amplitude values were found to have a normal distribution. The motor latency, as well as the medial plantar latency and lateral plantar sensory amplitude and latency data was found to have a non-normal positively skewed distribution. The motor latency data was transformed with a square root as this most closely approximated a normal curve. A natural logarithm transformation was completed on the medial and lateral plantar sensory amplitude and latency values to produce the most normal curve for this data. All reliability calculations for skewed data were computed on normalized data.

Eighty participants were present for baseline assessment, and 39 participants were tested in four test sessions over 6 months. One outlier was removed from the final analysis, so that 79 participants were included in the baseline assessment. Participant characteristics are presented separately for both groups (Table 1).

**Table 1:** Participant description

	N = 79	N = 38
Age (years)	23.6 ± 2.7	23.4 ± 2.6
Height (cm)	165.6 ± 6.4	166.0 ± 6.2
Weight (kg)	64.6 ± 12.4	62.8 ± 10.8
BMI	23.4 ± 3.4	22.7 ± 3.3

Anthropometric characteristics (mean ± SD) at baseline for 79 participants (normative data analysis) and 38 participants returning for reliability testing.

Tibial motor nerve recording from the FHB yielded a clear and consistent initial negative deflection from baseline, as did all motor waveforms included in this study. Motor and sensory mean ± SD nerve values, as well as range and upper and lower limits of normal (± 2 SD), first, second, third quartiles and the 10^th ^and 90^th ^percentiles at baseline are presented in Table 2.

**Table 2:** Tibial normative data

	Mean ± SD	Range	Upper/lowerlimit	Q1	Q2 Median	Q3	10 percentile	90 percentile
Tibial Motor (FHB)								
Latency (ms) 11.5 cm distance	4.1 ± 0.9	2.9 - 7.0	< 5.9	3.5	3.9	4.6	3.2	5.5
Amplitude (mV)	8.0 ± 3.0	1.8 - 18.4	> 2.0	5.5	8.0	10.0	3.8	11.4
Velocity (m/s)	45.6 ± 3.4	39.4 - 54.4	> 39.0	43.1	45.4	48.3	41.0	50.0
Medial plantar Sensory								
Latency (ms) 11.5 cm distance	2.8 ± 0.3	2.0 - 3.7	< 3.8	2.6	2.7	2.9	2.5	3.2
Amplitude (μV)	26.7 ± 10.1	6.6 - 40.9	> 6.5	20.3	26.8	35.4	13.7	43.1
Velocity (m/s)	41.4 ± 3.5	32.3 - 48.4	> 34.4	39.1	42.2	43.9	35.8	46.0
Lateral plantar sensory								
Latency (ms) 14.0 cm distance	3.2 ± 0.5	2.3 - 6.0	< 4.2	2.9	3.1	3.3	2.7	3.7
Amplitude (μV)	13.3 ± 4.7	6.0 - 36.8	> 3.9	10.3	13.3	19.6	7.9	25.7
Velocity (m/s)	44.3 ± 4.0	34.5 - 52.7	> 36.3	41.8	45.0	46.9	39.2	49.5

Normative data for nerve conduction parameters (mean ± SD, range) with clinically relevant upper and lower limits presented as the value 2 SD above/below the mean.

Footnote: Distances are rounded to the nearest 1/2 centimeter (cm). Q1 = first quartile, Q2 = second quartile, Q3 = third quartile.

The serial means ± SD as well as measures of reliability (ICC) and variability including the variable coefficient of variation and model coefficient of variation values are presented in Table 3. ICC calculations were completed on the raw data with the exception of the motor latency, as well as the medial and lateral plantar sensory amplitude and latency values in which the calculation was completed on the transformed values.

**Table 3:** Tibial reliability data

			Mean ± SD	ICC (3,1)	P value	MDC	CV variable	CV model
Motor Flexor hallucis brevis	Latency (ms)	Baseline	4.2 ± 0.9	0.2	0.003		0.16	
		8 wks	4.2 ± 0.7			1.63	0.14	0.24
		16 wks	4.3 ± 0.6			1.38	0.14	0.14
		24 wks	4.3 ± 0.6			1.38	0.13	0.12
	Amplitude (mV)	Baseline	7.9 ± 2.9	0.5	< 0.001		0.36	
		8 wks	8.4 ± 2.7			4.97	0.32	0.31
		16 wks	9.3 ± 2.7			4.69	0.29	0.23
		24 wks	8.4 ± 2.8			4.69	0.34	0.20
	Velocity (m/s)	Baseline	45.3 ± 3.1	0.4	< 0.001		0.07	
		8 wks	44.5 ± 2.9			4.97	0.06	0.06
		16 wks	44.1 ± 2.7			4.69	0.06	0.05
		24 wks	43.9 ± 2.7			4.86	0.06	0.04
Medial plantar sensory	Latency (ms)	Baseline	2.8 ± 0.3	0.5	< 0.001		0.10	
		8 wks	2.8 ± 0.3			0.52	0.10	0.09
		16 wks	2.8 ± 0.3			0.52	0.10	0.08
		24 wks	2.8 ± 0.3			0.52	0.10	0.08
	Amplitude (μV)	Baseline	27.7 ± 9.8	0.5	< 0.001		0.35	
		8 wks	26.7 ± 9.4			16.34	0.36	0.31
		16 wks	28.7 ± 11.1			17.22	0.39	0.27
		24 wks	26.9 ± 10.3			17.91	0.39	0.31
	Velocity (m/s)	Baseline	41.0 ± 3.2	0.4	< 0.001		0.08	
		8 wks	45.3 ± 4.6			8.58	0.10	0.10
		16 wks	44.3 ± 4.0			7.81	0.09	0.09
		24 wks	43.8 ± 4.4			8.39	0.10	0.08
Lateral plantar sensory	Latency (ms)	Baseline	3.2 ± 0.3	0.3	< 0.001		0.10	
		8 wks	2.9 ± 0.3			0.64	0.10	0.15
		16 wks	2.9 ± 0.3			0.64	0.10	0.10
		24 wks	2.9 ± 0.3			0.64	0.10	0.09
	Amplitude (μV)	Baseline	15.8 ± 7.1	0.2	0.007		0.45	
		8 wks	14.9 ± 5.1			10.90	0.36	0.44
		16 wks	14.9 ± 5.0			10.30	0.34	0.34
		24 wks	15.2 ± 5.8			11.60	0.39	0.40
	Velocity (m/s)	Baseline	43.6 ± 3.4	0.4	< 0.001		0.08	
		8 wks	47.5 ± 4.1			8.77	0.09	0.09
		16 wks	46.4 ± 4.4			9.38	0.10	0.09
		24 wks	46.8 ± 4.6			9.83	0.10	0.08

Serial tibial nerve values (mean ± SD) for participants completing the reliability study (baseline, 8 wks, 16 wks, 24 wks) with intraclass correlation coefficient (ICC) values, corresponding P values, variable coefficient of variation and model coefficient of variation (CV) values

Tibial motor (FHB) latency values demonstrated up to 16% variability between participants and 24% variability within participants. The motor FHB latency values demonstrated an ICC value of 0.2 *(P *= 0.003). The medial plantar sensory latency variability maximum values were 10% between participants and 9% within participants. The medial plantar sensory latency study also demonstrated good reliability with an ICC value of 0.5 (*P *< 0.001). The lateral plantar sensory latency coefficient of variation values displayed up to 10% variability between participants and a maximum of 15% variability within participants. The lateral plantar sensory latency ICC value was 0.2 (*P *= 0.003).

Tibial motor FHB amplitudes demonstrated variability ranges up to 36% between participants and 31% within participants. The ICC value for the tibial FHB motor amplitude revealed a correlation value of 0.2 (*P *= 0.001). Medial plantar sensory amplitude values demonstrated variability between and within participants up to 39% and 31% respectively. Medial plantar sensory amplitude studies displayed an ICC value of 0.5 (*P *< 0.001). The lateral plantar sensory amplitude revealed variability ranging up to 45% between participants and 44% within participants and an ICC value of 0.2 (*P *= 0.002).

Tibial motor FHB velocity coefficients of variation revealed up to 7% variability between participants and 6% variability within participants. The tibial motor FHB velocity ICC value was 0.4 (*P *< 0.001). The medial plantar sensory conduction velocity values revealed up to 10% variability both between and within participants. The medial plantar sensory velocity reliability coefficient was 0.4 (*P *< 0.001). The lateral plantar sensory conduction velocity calculations demonstrated up to 10% variability between participants and 9% variability within participants. The lateral plantar sensory velocity ICC value revealed a value of 0.4 (*P *< 0.001).

## Discussion

Our findings indicate that tibial motor nerve recording from the FHB is practical and reproducible. In addition, we have presented reference values for this approach in a young adult female population. We have further added to the body of normal values for orthodromic medial and lateral plantar sensory studies. The American Association of Neuromuscular and Electrodiagnostic Medicine recommends both motor and sensory studies for the evaluation of posterior tarsal tunnel syndrome, making these tests particularly relevant [13].

Felsenthal et al reported recording the medial plantar motor branch from the FHB muscle, however they reported only latencies in a small number of participants [20]. Our recording techniques differed from Felsenthal's description in that we recorded from the lateral head of the FHB, while he described recording from the medial head. We noted a clearer waveform with recording from the lateral head, possibly due to positioning further from the abductor hallucis. When using our described technique for tibial motor recording, the recording electrode placement over the FHB needs to be located immediately adjacent to the lateral side of the flexor hallucis longus tendon to ensure placement is over the FHB and not neighboring intrinsic muscles. The two heads of the FHB surround the flexor hallucis longus tendon. Neighboring intrinsic muscles would most likely be the medial plantar motor innervated first lumbrical, or the lateral plantar motor innervated oblique head of the adductor hallucis muscle [1]. Our recording location just lateral to the flexor hallucis longus tendon maintains positioning over the first metatarsal and lateral head of the FHB. This location would be distal to the first lumbrical which although it is medial plantar innervated is a very small muscle and not likely to be large enough to impact the recording from the FHB. The oblique head of the adductor hallucis lays deep and lateral to the FHB muscle, so that if the recording electrode is positioned immediately next to the flexor hallucis longus tendon, it is most likely to be recording from the more superficial FHB. The first dorsal and plantar interosseous muscles are also nearby, but are deep to the adductor hallucis so that recording from the interossei would be unlikely with our montage. In addition, we have observed that supramaximal stimulation of the medial plantar motor branch in the foot 5 cm proximal to the recording electrode over the FHB produces the same waveform amplitude and morphology as does stimulation of the tibial nerve proper at the ankle. This would indicate that this recording site is medial plantar nerve supplied; ruling out the possibility of recording from the lateral plantar innervated oblique head of the adductor hallucis.

When examining a patient with suspected tarsal tunnel syndrome, tibial motor recording from the FHB may be superior to the more commonly used abductor hallucis. This is especially significant, since the abductor hallucis has been noted to produce technical difficulty, leading to error in assessing tarsal tunnel conduction [17]. We did note a clear consistent initial negative deflection from baseline with recording from the FHB in all participants, which would improve the ability to obtain an accurate nerve conduction velocity in a short segment such as through the tarsal tunnel. We attempted to approximate a clinical situation, however this did lead to some limited amount of control particularly in that foot size variations produced some varying degree in distances used for the latency values. The standard distance for motor recording electrodes to be placed from the distal stimulating electrode is 8 cm [2,14,16], however recording from such a distal point over the FHB makes an 8 cm distance impractical in that stimulation would then be applied in the foot rather than at the ankle. Our established 12 cm distance for the tibial motor and medial plantar sensory and our established lateral plantar sensory distance of 14 cm required some variation to produce an optimal response in some participants. The motor and sensory latency values were quite stable over time indicating that although there was some movement of electrodes to achieve the best waveform, a significant deviation was not needed in the majority of participants. The mean distance from the motor and medial plantar sensory recording electrodes to the distal stimulation site was 11.5 cm. The mean distance from the lateral plantar sensory recording electrodes and the distal stimulation site was 13.9 cm.

Calipers are sometimes used rather than tape measures to measure irregular surfaces such as in the arm and axilla [16,29]. We chose a tape measure technique for determining all distances including the foot, as we felt it would more accurately assess the length of the tibial nerve in the medial ankle area. In a cadaver comparison of surface measures of the radial nerve, it was reported that surface tape measures more accurately correlated with the length of the dissected nerve, than did caliper measurements [29]. In addition, large normative tibial motor studies with recording from the abductor hallucis and flexor digiti minimi also used a tape measure to assess distances in the foot [14,30]. The variation in distances used to produce an optimal waveform is a limitation of our study, however it may indicate that motor latency to the flexor hallucis brevis will be difficult to standardize clinically so that conduction velocity through the tarsal tunnel may be the most ideal measure of distal motor function. It may also be particularly useful to compare distal motor latency with the uninvolved side when possible.

It is difficult to compare our sensory latency and conduction velocity values with other studies that used a similar recording montage. The American Association of Neuromuscular and Electrodiagnostic Medicine recommends the use of peak medial and lateral plantar values in the evaluation of potential posterior tarsal tunnel syndrome [13]. We also chose to use peak sensory latency values as did Loseth et al [25], while Iyer et al [21], Galardi et al [31], and Antunes et al [24] reported latency and conduction velocity values from the onset of the sensory waveform instead of the peak of the waveform. Not all normative studies maintained temperature control, making a direct comparison with our results difficult [21]. Loseth et al reported warming the limb if surface temperature dropped below 27 degrees Celsius [25]. Buschbacher reported warming the limb and monitoring temperature such that all limbs were greater than 31 degrees Celsius in normative tibial and peroneal motor studies. No range of actual recorded temperatures was reported [14,25,30,32]. Antunes et al evaluated sensory potentials in an orthodromic fashion similar to our protocol and reported establishing a fixed distance for medial and lateral plantar sensory studies at 14 cm with repositioning of the electrodes as needed to obtain an adequate potential. They did not further report their range of distances in the event electrodes were repositioned [24]. Ponsford et al [33] also used a similar orthodromic technique, but did not report their distances. Ponsford et al [33], Antunes et al [24] and Loseth et al [25] did not make note of a caliper versus tape measure technique. Utilizing the same distance for medial and lateral plantar sensory studies would make a direct comparison between the medial and lateral plantar sensory latency valuable, however in some individuals it may be difficult to obtain the optimal waveform at the same distance. It may be that due to callous formation and large variations in foot size, sensory nerve conduction velocities are a more accurate measure of sensory nerve function in the foot than latency values.

Our sensory amplitude to SD ratios were similar to those of previous normative studies [19,23,27,33] and sensory amplitudes are reportedly highly variable over time [16]. We also recorded larger medial and lateral plantar sensory amplitudes than reported in previous studies using the same orthodromic mixed nerve technique [21,25,33]. The larger amplitude values may be due to the younger participant population in our study as compared with other published studies. Many authors note that amplitude and velocity does decrease with age, and that tibial sensory values are difficult to obtain in those over 60 years of age [14,25,31]. Body mass index (BMI) may also be a factor in nerve conduction studies, with Bushbacher reporting a decrement in recorded sensory and mixed nerve amplitude values in those with higher BMI values [34]. Our studied population had a relatively low mean BMI with little variance. This may further explain why our sensory amplitude values are larger than those published previously.

A moderate degree of variability was noted in our reported tibial (FHB) motor as well as medial plantar and lateral plantar sensory amplitudes both within and between participants. All amplitude parameters did, however, demonstrate a statistically significant correlation over time. Latency parameters for all measures also revealed significant correlations indicating that they are reliable measures as well, even though some variation was noted in the distance needed to produce an optimal potential. The velocity values were the most stable measures with a model motor coefficient of variability between 4 and 6%. The medial and lateral plantar conduction velocity model values were a little more varied with values that ranged from 8-10% and 8-9% respectively.

We observed low ICC values for a number of our measures. The ICC is the most robust and widely used statistic to describe reliability of clinical tests. However, the ICC is limited by the fact that its value is subject to the variability of the sample used to construct it. The ICC will be low if the variability of the sample is too large, but is also susceptible to being low if the sample from which it is calculated has low variability (the sample is very homogenous). Despite a low ICC value for several of our measures (tibial motor velocity, medial plantar sensory amplitude and velocity, lateral plantar latency and velocity), the low variability of an individual's measurements over time allows some latitude to be exercised in interpreting appropriate statistical reliability. In other words, given the low temporal variability of these measures, we can be confident in identifying differences in these measures over time, despite a low ICC value. Similarly, the between participant variability in our sample was quite low for latency (0.10-0.16) and velocity (0.06-0.10) measures for both motor and sensory studies, though the ICC values for these measures ranged between 0.20 and 0.50. The fact that variability between individuals consistently falls below our observed ICC values demonstrates the ability of these values to serve as appropriate normal values for this population of individuals.

The participants in this study were women between the ages of 18 and 35, so that results may not be generalizable to other ages and genders. Future studies should include trans-tarsal conduction values, larger age ranges, greater sample size, and greater geographic diversity and should include men as well as women. A larger sample size would allow for a calculation of percentiles among different age groups as suggested by Obrien et al as a more effective way of reporting normal values [35].

## Conclusion

Our study results indicate that motor recording from the FHB produces a clear and consistent initial negative deflection from baseline, allowing for an accurate assessment of nerve conduction velocity across the tarsal tunnel. This may make recording from the FHB preferable in assessing posterior tarsal tunnel syndrome when technical challenges occur with abductor hallucis recording. The motor recording from the FHB, as well as the orthodromic medial and lateral plantar sensory values have acceptable intra-rater reliability and variability.

## Competing interests

The authors declare that they have no competing interests.

## Authors' contributions

KG carried out the nerve conduction studies. KG performed statistical analysis. All authors participated in the design and coordination of the study. All authors helped draft the manuscript, and have read and approved the final manuscript.
